# Effect of mineral excipients on processing traditional Chinese medicines: an insight into the components, pharmacodynamics and mechanism

**DOI:** 10.1186/s13020-021-00554-8

**Published:** 2021-12-24

**Authors:** Yan Liu, Xiaojie Li, Cai Chen, Aijing Leng, Jialin Qu

**Affiliations:** 1 Department of Traditional Chinese Medicine, The First Affiliated Hospital of Dalian Medical University, No. 222, Zhongshan Road, Dalian, 116011 China; 2 Clinical Laboratory of Integrative Medicine, The First Affiliated Hospital of Dalian Medical University, No. 222, Zhongshan Road, Dalian, 116011 China; 3 Institute (College) of Pharmacy, Dalian Medical University, No. 9, South Road of Lvshun, Dalian, 116044 China; 4 Institute (College) of Integrative Medicine, Dalian Medical University, No. 9, South Road of Lvshun, Dalian, 116044 China

**Keywords:** Mineral excipients, Processing, Traditional Chinese medicine (TCM), Components, Pharmacodynamics, Mechanism

## Abstract

Traditional Chinese medicines are an important class of natural products mainly derives from animals, plants and minerals, most of which need to be improved and processed before clinical use due to their own hard texture, impurities or toxicity. As an important part of solid excipients, mineral excipients that contain some metal elements play indispensable and unique roles in the pretreatment process of traditional Chinese medicine. However, deficiency of holistic understanding of the effect of mineral excipients hinders their application and development. This article reviews several mineral excipients including alumen, talci pulvis, soil, soda lime, halloysitum rubrum and cinnabaris systemically. Their processing significance on traditional Chinese medicines were revealed from components, pharmacodynamics and mechanism aspects. Furthermore, prospect and problems including processing technologies, quality standards of mineral excipients and processing mechanism were put forward. This review supply comprehensive information for better and scientific usage of mineral excipients in processing traditional Chinese medicines.

## Introduction

Traditional Chinese medicines (TCM) are an important class of natural products mainly derives from animals, plants and minerals, most of which need to be improved and processed before clinical use due to their own hard texture, impurities or toxicity, and the pretreatment process is called Chinese medicine processing. In the process of processing, the active ingredients such as alkaloids, glycosides, volatile oils, tannins, organic acids, proteins, amino acids, sugars and inorganic compounds will be affected. Generally, the processing methods of TCM are mainly divided into cleaning, cutting, heating, and adding excipients. Cleaning is used to remove the impurities and non-medicinal parts. Cutting contributes to the decoction of effective components through changing the shape of medicinal materials. Heating is applied to changing the texture and efficacy, correcting the bias, improving the decoction of effective ingredients as well as reducing toxicity and side effects through the procedures of frying, calcining, steaming, boiling, etc. [1]. Adding excipients could not only change the nature, flavor, action and meridian tropism of crude drugs, but also enhance the efficacy and reduce or diminish the toxicity and side effects. Among which, the application of excipients that possesses a long history has reflected the flexibility of clinical medication. And the role in processing medicinal materials is also variable accompanied by the difference between varieties, properties and functions of excipients.

Overall, processing excipients could be divided into liquid excipients and solid excipients. Yellow rice wine, vinegar, salt-water and refined honey are the commonly used liquid ones. Yellow rice wine could promote the upward direction, enhance the effect of blood-activating and stasis-resolving medicinal in invigorating blood circulation, moderate the cold and cool of some herbs with cold-nature [2].Vinegar could reinforce the effect of liver-soothing, dissipating blood stasis and relieving pain of TCM synergistically. Besides, several TCM processed with salt-water could conduct the drug to the kidney meridian and strengthen the efficacy on lower-jiao diseases. Refined honey could change drugs become sweet and sluggish, enhance the effects of benefiting Qi, moistening lung, relieving a cough and stopping pain and dysentery. In addition, ginger juice, licorice juice, black bean juice, bile, lanolin and sesame oil that are used for specific herbs also belongs to the categories of liquid excipients. As for solid excipients, rice could generally reduce the odor and toxicity of animal drugs and increase the role of some tonic drugs in improving the interior and benefiting Qi. Bran could absorb excessive volatile oil and reduce irritation of herbs. Sand helps the medicine to be friability due to homogeneous heating and reduces the toxicity as well. Except for the above commonly used ones, some mineral excipients that not only acts as medicinal materials but also enhance the therapeutic effect and reduce the toxicity were also applied in the process of TCM.

In the Chinese Pharmacopoeia (ChP, 2020 edition), 4 kinds of TCM that processed with mineral excipients has been included. Pinelliae Rhizoma Praeparatum Cum Alumine (Qingbanxia), Pinelliae Rhizoma praeparatum cum Zingibere et Alumine (Jiangbanxia), and Rhizoma Pinelliae Praeparatum (Fabanxia) that derive from the raw *Pinellia ternate* (Thunb.) Breit. were processed with alumen, ginger juice and soda lime as adjuvant materials, respectively. Arisaematis rhizoma preparatum (Zhitiannanxing) that derives from the *Arisaema erubescens* (Wall.) Schott, *Arisaema heterophyllum* Bl. or *Arisaema amurense* Maxim. is processed with alumen. Besides, Typhonii Rhizoma (Baifuzi) and Hirudo (Shuizhi) that were separately processed with alumen and talci pulvis has also been included as one of the main processing methods, although relative standards have not formed. Meanwhile, some provinces have promulgated their own standards. For example, Atractylodis Macrocephalae Rhizome (Baizhu) and Paeoniae Radix Alba (Baishao) that stir-fried with soil have been included in the standard for preparation of Chinese herbal pieces in Beijing (2008 edition). Coicis Semen (Yiyiren) stir-fried with soil has been included in the standard for cut crude drug of TCM in Yunnan Province (2005 edition). Moreover, several processed products including Angelicae Sinensis Radix (Danggui) stir-fried with soil, Ophiopogonis Radix (Maidong) mixed with Cinnabaris had also been included in provincial processing specification, even though there is still no formal standards to follow (Table 1). The aforementioned information suggests the essential role of mineral excipients in the field of TCM processing. However, their research status and processing mechanism has not been systematic summarized, which hinders their application and development.

**Table 1:** Ancient and modern comparison of processing methods on TCM with mineral excipients

TCM		Modern processing				Ancient processing
		Chinese pharmacopoeia (2020 edition)	Provincial processing specification			
			Processing method	Provinces or Municipalities	Edition	
Pinelliae Rhizoma (Banxia)	Pinelliae Rhizoma praeparatum Cum Alumine	Take Banxia → soak in 8% alumen → boil thoroughly, reduced sensation of numb tongue → wash → cut into thick slices → dry	–	–	–	Take Banxia → soak in alumen for 7 days → dry *Shengjizonglu*(《聖濟總錄》)
	Pinelliae Rhizoma Praeparatum cum Zingibere et Alumine	Take Banxia → soak in water → boil thoroughly → add ginger and alumen → boil → take out → dry	–	–	–	Take Banxia → boil in alumen → add ginger → make into paste *Bencaomengquan* (《本草蒙筌》)
	Rhizoma Pinelliae Praeparatum	Take Banxia → soak in water → boil thoroughly → add licorice boiled liquid → add lime water → soak and maintain pH12 → golden yellow section, reduced sensation of numb tongu → wash → dry	–	–	–	Take Banxia → add boiled lime water → stir → clarify it and discard the residu *Bencaogangmushiyi*(《本草綱目拾遺》) Take Banxia → add lime water → soak 2–3 days → wash → add alumen → soak → wash → dry *Yaoxingcuping* (《藥性粗評》)
Arisaematis Rhizoma (Tiannan Xing)	Arisaematis rhizome preparatum	Take Tiannanxing → soak in water → change the water 2 or 3 times a day → white foam appeared → soak in alumen for one day → change the water → sensation of numb tongue is reduced → add ginger and alumen → boil thoroughly → air to 40% ~ 60% dry → cut into slice → dry				Take Tiannanxing → soak in alumen for 3 days → dry *Bencaobeiyao*(《本草備要》) Take Tiannanxing → soak in alumen → cleaning 7 times → boil *Shengjizonglu*(《聖濟總錄》 Take Tiannanxing → add lime → stir-fry *Xianshoulishangxuduanmifang* (《仙授理傷續斷秘方》)
Typhonii Rhizoma (Baifuzi)		Take Baifuzi → soak in water → change the water 2–3 times a day → sticky foam appears → soak in alumen for1 day → change the water → sensation of numb tongue is reduced → add ginger and alumen → boil → boil thoroughly → air to 60% ~ 70% dry → cut into thick slices → dry	Take Baifuzi → soak in water for 15 ~ 20 days → change the water 2–3 times a day → sticky foam appears → change the water → soak in alumen for 3 days → change the water → sensation of numb tongue is reduced → add ginger and alumen → boil → boil thoroughly → air to 60%- 70% dry → cut into thick slices → dry	Beijing	2008	
Hirudo (Shuizhi)		Take stir-fry talci pulvis → add Shuizhi → until slightly puffed	Take stir-fry talci pulvis → add Shuizhi → until light yellow, slightly bulging → take out	Jiangxi	2008	–
Myristicae Semen (Roudoukou)		–	Take stir-fry talci pulvis → add Roudoukou → until the surface is slightly bulgy and yellowish → take out	Beijing	2008	–
Asini Corii Colla (Ejiao)		–	Take diced Ejiao → add talci pulvis → stir-fry → until bulged into beads without a soft yolk → take out	Hunan	2010	–
Angelicae Sinensis Radix (Danggui)		–	Take Danggui → add soil → stir-fry → until earth-yellow → sift the soil away → cool	Hunan	2010	Take Danggui → add soil → stir-fry *Depeibencao*(《得配本草》)
Atractylodis Macrocephalae Rhizome (Baizhu)		–	Take stir-fry soil → add Baizhu → stir-fry → until surface is the color of soil, fragrant → sift the soil away → cool	Shandong	2012	Take Baizhu → add soil → stir-fry → until brown → sift the soil away *Bencaomengquan*(《本草蒙筌》) Take Baizhu → add soil → stir-fry *Qianjinyifang*(《千金翼方》) & *Bencaocongxin*(《本草从新》)
Paeoniae Radix Alba (Baishao)		–	Take Baishao → add soil → stir-fry → until yellowish brown → sift the soil away → cool	Tianjin	2018	–
Coicis Semen (Yiyiren)		–	Take stir-fry soil → add Yiyiren → until surface is the color of soil, fragrant → sift the soil away → cool	Yunnan	2005	Take Yiyiren → add soil → stir-fry → until surface is yellow → boil → grind in paste form *Youhuanjiwen*(《遊宦紀聞》)
Citri Reticulatae Pericarpium (Chenpi)		–	Take stir-fry soil → add Chenpi → stir-fry → until surface is the color of soil with focal spot → sift the soil away → cool	Shandong	2012	–
Ophiopogonis Radix (Maidong)		–	Take Maidong → spray a little water → add cinnabaris → mix well → dry	Hunan	2010	Take Maidong → add cinnabaris *Bencaobiandu* (《本草便讀》)
Polygalae Radix (Yuanzhi)		–	Take Yuanzhi → spray a little water → moisten for	Gansu	2009	–

**Fig. 1:**
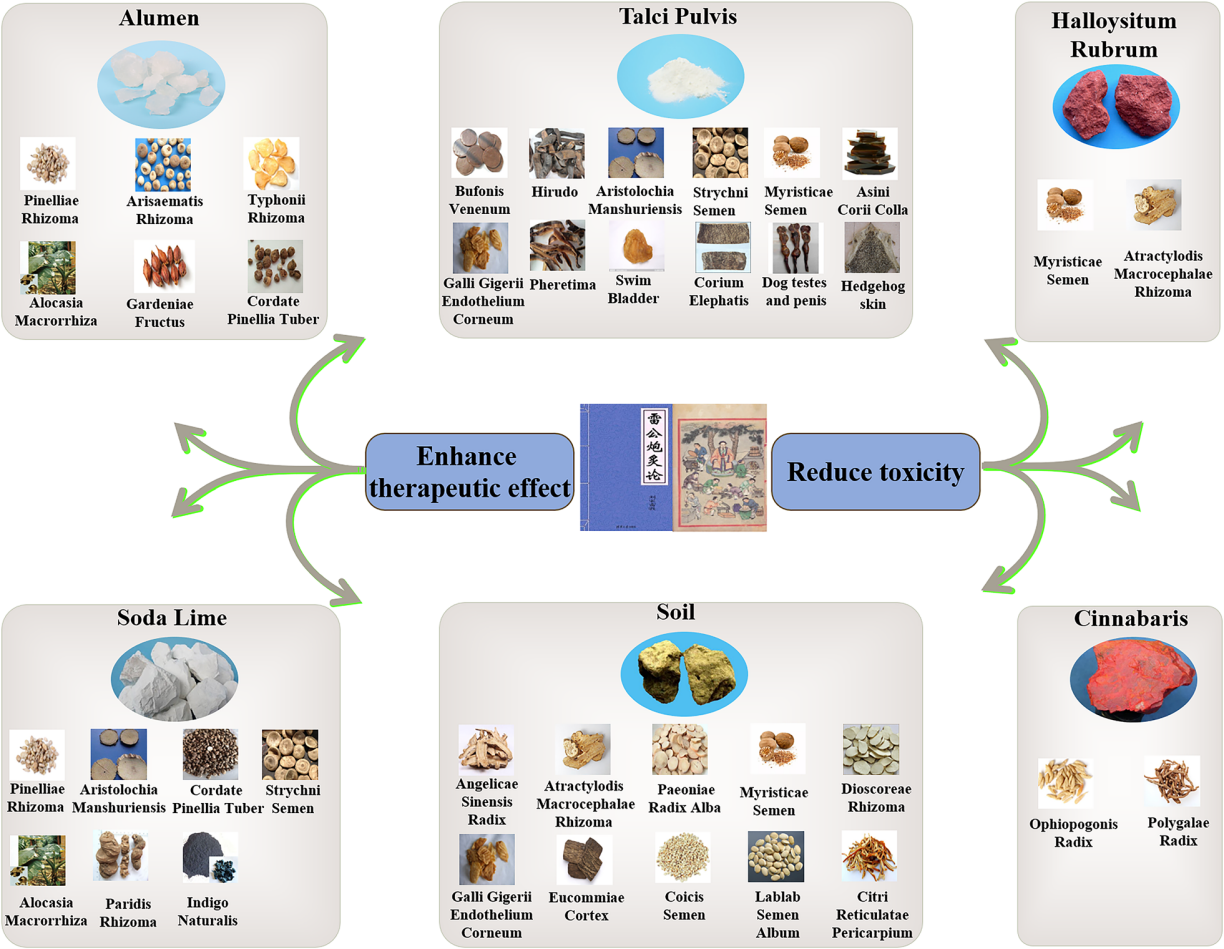
Effect of mineral excipients on processing TCM

In this paper, the application of several mineral excipients in the processing of TCM is reviewed by sorting out the existing literature reports (Fig. 1, Table 1). Their processing significance on TCM were revealed from components, pharmacodynamics and mechanism aspects, which provides references for better usage of TCM.

### Effect of mineral excipients on the processing of TCM

#### Alumen

**Fig. 2:**
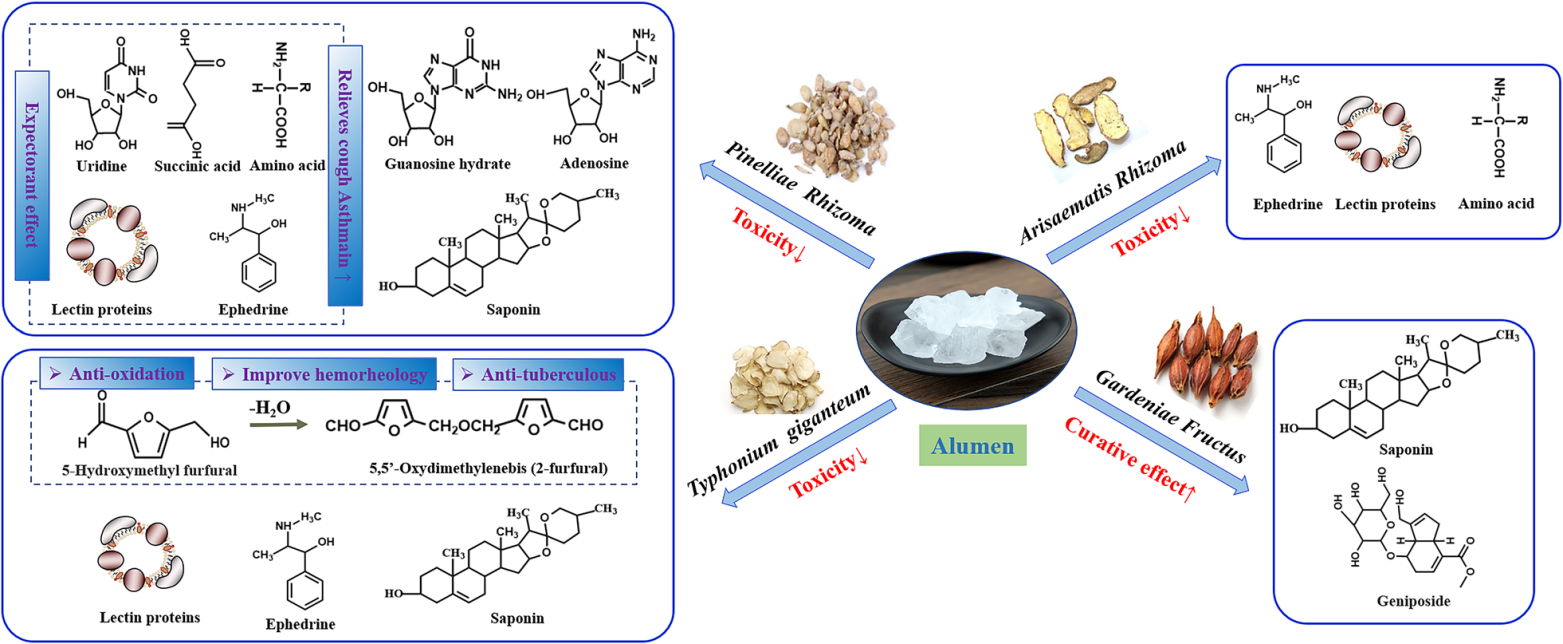
Effect of Alumen on the processing of TCM

Alumen is a kind of sulfate mineral alumen stone, which is processed and refined from alunite. It can be used as an external medicine to exert the effects of detoxification, insecticidal, moisturizing and relieving itching as well as oral administration for diseases such as bleeding and diarrhea. Apart from the above physical and biological actions as a TCM, the alumen itself is often used as a processing excipient to reduce toxicity and increase efficacy. Banxia, Baifuzi and Tiannanxing are typical TCM processed with alumen (Fig. 2).

The processing method of Banxia and Tiannanxing with alumen could be traced back to the *Song Dynasty*. The former has been recorded in many classical medical books in China, such as *Taipinghuiminhejijufang*(《太平惠民和剂局方》), *Shengjizonglu*(《聖濟總錄》) and *Baoqingbencaozhezhong*(《宝庆本草折衷》), while the latter was first documented by *Shengjizonglu* [3–5]. Besides, standards for the processed products of Baifuzi with alumen have been included in the *ChP* and 26 provincial processing specifications, which is gradually developed on the basis of previous licorice and ginger processing. So far, boiling or steaming Baifuzi with alumen or ginger has become the universal processing means [6].

#### Pinelliae Rhizoma

Pinelliae Rhizoma, commonly known as 'Ban-xia' in Chinese, is botanically from the dried tuber of *Pinellia ternata* (Thunb.) Breit., which has been used to remove dampness and dissipate phlegm, reduce adverse reactions, stop vomiting as well as eliminate swelling and scattered nodules. Furthermore, it is also a famous poisonous plant, and its toxicity is attributed to alkaloids and toxic proteins. Lectin is the most important toxic component of Banxia. And it has been confirmed to have obvious toxic damage to many organs, such as liver, digestive tract, kidney and heart. Interestingly, when verifying the toxic effect of Banxia on stomach and digestive tract, some researchers found that 50 mg/mL of Banxia could inhibit gastric nerve activity in rats, while it did not show any inhibitory effect when processed with ginger juice. Moreover, it also exhibits obvious reproductive toxicity, which lead to the termination of pregnancy and fetal deformity or death. Animal experiments showed that intragastric administration of Banxia powder (9 g/kg) and Banxia decoction (30 g/kg) could increase early embryo mortality by 85.7% and 50.0%, respectively [7–9]. Similarly, Banxia no longer causes vomiting and hoarseness in experimental animals after the processing with alumen [10].

As one of the classic herbs processed by alumen, the contents of alkaloids, organic acids, nucleotides, polysaccharides, proteins, amino acids and inorganic elements in Banxia were mainly changed. Ephedrine, a major alkaloid constituent that relieves cough and asthma, its content increases after processing due to nonvolatile and stabilization of ephedrine salt formed by the action of acidic alumen water [10–12]. In addition, there are also cases where the content of alkaloid is lower than that of raw one by different processing technology [10, 13]. For example, a significant decrease in the content was observed in other alkaloids including guanosine, uridine and adenosine compared with the crude product, which may be related to the breaking and decomposition of glycosidic bond by dissolving in water and alumen [10, 11, 13–16]. Moreover, changes of these three alkaloid components could affect the expectorant effect of Banxia. Meanwhile, content of organic acid such as succinic acid in processed Banxia was usually higher than that of raw products [11]. While it was also reported that their content was slightly lower or similar to that of the raw one. The main reason was that the total organic acid was dissolved in water, and the difference would occur owing to the different processing parameters [10].

Detoxification is also one of the main purposes of Banxia processing, calcium oxalate needle crystal with special crystalline and lectin protein are main toxic components in it. After being processed with alumen, the toxic needle crystal dissolved and corroded, and the agglutinin protein degraded and denatured, so the toxicity is reduced [10, 14, 17–23]. However, the lectin protein is also an effective component of Banxia with anti-tumor effect [14]. Thus, explore how to moderately processing Banxia with alumen has important implications.

The content of amino acids in processed Banxia was higher than that in the raw one [13]. It was also reported that the quantity and species of amino acids, sugars, fatty acids and phytosterols had no change, while the content was much lower than that of crude drugs [24]. The sugar content decreased significantly because of its dissolution in water. In addition, the strong acid action of aluminum can degrade triacylglycerol into diacylglycerol, monoacylglycerol and glycerol [24]. Some trace elements also changed, the content of zinc, magnesium and iron in inorganic elements increased sharply accompanied by the introduction of nickel at the same time [10, 13]. Some studies still pointed out that the reducing sugar content of Qingbanxia is higher than that of the raw one, conversely, the total sugar content is lower [10].

Comparative investigation of curative effect and aluminum residue after alum processing is also worthy of attention. The content of succinic acid and alkaloids increased after the preparation of Banxia by alumen, which was beneficial to improve several therapeutic effects, such as antitussive, antiasthmatic, anti-ulcer, etc. [11]. And alumen itself has the effect of dispelling wind phlegm, which helps enhance the therapeutic effect of Banxia. Besides, decrease of some proteins, polysaccharides and nucleotides can also affect the anticancer effect of Banxia, but the irritant toxicity of raw one is significantly reduced after being processed with alumen. It has been reported that there is aluminum residue after alum processing in Banxia. However, some aluminum ions are converted into stable small toxic granular aluminum due to the intense boiling conditions, which reduces the direct toxicity of aluminum in Banxia to human body [25] (Table 2).

**Table 2:** Processing effect of mineral excipients on Traditional Chinese medicine from components, pharmacodynamics and mechanism aspects

Adjuvant	Traditional Chinese medicines	Chemical ingredients	Pharmacodynamic	Processing mechanism	Refs.
Alumen	Pinelliae Rhizoma	(**↑**) ephedrine, organic acid, succinic acid, amino acid, trace elements (Zn, Fe, Mg, Ni) (**↓**) alkaloids (ephedrine, guanosine, adenosine, uridine), toxic constituents (lectin protein, calcium oxalate needle crystal), succinic acid, carbohydrate, fatty acid, phytosterol, amino acid, triacylglycerol	(↓) irritant toxicity, anticancer (↑) remove dampness and dissipate phlegm	(1) The needle crystal of calcium oxalates was corroded and dissolved by alum water from two aspects: a) sulfate ion generated from aluminum potassium sulfate; b) reaction of insoluble calcium oxalate under strong alkali solution (2) The destroyed lectin protein, changed and degraded the peptide sequence in alum water	[10–25]
	Arisaematis Rhizoma	(**↑**) Trace element Fe (↓) Amino acids, alkaloids, trace elements (Zn, Ca), lectin protein, calcium oxalate needle crystal	(↓) irritant toxicity	(1) Calcium oxalate needle crystal was dissolved in acidic alum solution; alum was hydrolyzed into aluminium hydroxide gel in water; toxic component is absorbed by its own charge (2) The lectin protein was destroyed and degraded in alum water	[21–23, 27–29]
	Typhonii Rhizoma	(↑) 5-hydroxymethylfurfural, bis (5-formylfuryl) ether (↓) amino acids, alkaloids, nucleosides, polysaccharides, total flavonoids, lectin protein	(↓) irritant toxicity (↑) dispel wind and phlegm, antivirus, antioxidant, anti-tuberculosis, improve hemorheology	(1) Under acidic and heated conditions, 5-hydroxymethylfurfural was produced by thermal degradation and Maillard reaction from sugars and free amino acids (2) Salification reaction of alkaloids in Aconitum carmichaeli in acidic alum water makes form salts, which increases water solubility and causes loss (3) The lectin protein and calcium oxalate needle crystals were degraded and failed to work in alum water (4) Alum could fix protein, promote lipid synthesis and increase fat soluble components (5) Nucleosides were easily soluble in water, resulting in loss	[21, 22, 31–37]
	Cordate Pinellia Tuber	(↓) polar components		Potassium aluminum sulfate promotes the dissolution of polar small molecule compounds	[39]
	Alocasia Macrorrhiza		(↓) toxicity 、antipyretic effect		[38]
	Gardeniae Fructus	(↑) total iridoid glycosides, geniposide, crocin I and crocin II (when using Gardenia jasminoides with six edges) (↓) geniposide, crocin I, total iridoid glycosides	retention of active ingredients (↑) curative effect	Enzyme killing and glycosides preserving	[40–42]
Talci Pulvis	Myristicae Semen	(↑) volatile oil (myristicin, safrole, eugenol, isoeugenol), trace elements( Fe, Mn, Zn, Cu) (↓) volatile oil (myristicin, safrole), fatty oil, tannins	(↑) antidiarrheal (↓) intestinal sliding, toxicity	(1) Long time heating (2) The change of volatile oil content was caused by heat transfer rate, particle size and adsorption	[46–50]
	Hirudo	(↑) amino acids, trace elements(Ca, Mg), hypoxanthine, volatile constituents (terpinene, 4-methylphenol, cycloalfalfa, 3,7,11-trimethyl-1,6,10-dodecatriene-3-ol, 2,6,10,14-tetramethylpentadecane) (↓) toxic metals (Pb, Cd, Hg), trace elements (Zn, Mn, Cu, Fe), hirudin, protein, volatile constituents (5-hydroxy methylfurfural, nonanoic acid, undecanol, decanoic acid, dodecanal, peach aldehyde, 2-dodecanone, 2-tetradecanone, myristic aldehyde, tridecanoic acid, 2-tridecanone, 1,2-epoxyectadecane, myristic acid, 2-pentadecanone, 1-nonadecene, 2-heptadecanone)	(↑) reduce blood pressure,relieving asthma (↓) anticoagulant activity, coagulation and toxicity	Heat destroys proteins	[51–55, 109, 110]
Talci Pulvis	Bufonis Venenum	(↓) bufogenin, resibufogenin	(↓) irritant toxicity	Heat denaturates the protein	[56–58]
	Aristolochia Manshuriensis	(↓) aristolochic acid A	(↓) nephrotoxicity	High temperature degradation	[60]
	Strychni Semen	(↓) strychnine, brucine	(↓) toxicity	Uniform thermal degradation	[59]
	Galli Gigerii Endothelium Corneum	(↓) amino acid, protease and amylase	(↑) eliminating kidney stones, treating stranguria	Enhances the efficacy synergistically; makes the texture crisp loose and crisp; convenient to prepare and carry; correct odor and taste	[62]
	Asini Corii Colla	(↑) amino acid	(↑) hemostasis and blood production (↓) greasy nature, bad smell and side effects	(1) Convergence, adsorption and intestinal wall protection effect by the magnesium silicate contained in Talc powder; (2) The calcium salt in donkey hides gelatin was processed into calcium ion, which is convenient for the human body to absorb and increase the calcium content in blood; Calcium ion is a coagulant, which could reduce the penetration of blood cell and serum outside the blood vessel wall. It could enhance the effect of astringency, hemostasis and blood-enriching (3) Talc can heat the drug evenly and absorb a lot of chemical irritants and water	[63, 64]
	Swim Bladder	(↑) protein, 3,4,5-trimethoxytoluene, volatile constituents (α-gurulene, benzophenone, guaiacene, pristane, 2-decyltetraethylene oxide, 6,10,14-triyl-2-pentadecanone) (↓) volatile constituents (2-decennal, myristic aldehyde, cedrene, nerolidol, heptadiene, 2-pentadecanone, heptadecane, cyclopentadecane, diisobutyl phthalate,1-octadecene, ethyl palmitate)	The efficacy was not affected	The intermediate heat transfer body makes it easy to process the preparation	[55, 65]
	Corium Elephatis	(↑) amino acid		Heating makes the elephant skin foaming and crisp, which is conducive to the preparation	[66]
	Dog testes and penis	(↑) alcohol extract (↓) amino acid		Makes the texture crisp loose and crisp; convenient to prepare and carry; correct odor and taste	[67]
	Hedgehog skin	(↓) alcohol extract, amino acid			[68]
	Puerariae Lobatae Radix	(↑) total flavonoids	(↑) spleen-tonifying, antidiarrhea		[69]
	Hawksbill Shell	(↑) volatile constituents (3-methylbiphenyl, caryophyllene oxide, benzophenone, pristine, 2,6-di-tert-butyl benzoquinone, methyl palmitate) (↓) volatile constituents (cedrene, valencia tangerine, elemanol, gladiolene, β-eucalyptol, myristic aldehyde, 6,10, 14-trimethyl-2-pentadecanone, octadecane epoxide, eucalyptol)			[55]
	Pheretima		(↑) stranguria, heat-clearing, diuretic	Synergism of talci pulvis	[61]
	Angelicae Sinensis Radix	(↑) conifer ferulate,1-ethoxypropane,2,6-dimethyl benzene anthracene, organic acid (chlorogenic acid, formic acid, acetic acid), volatile constituents (butyraldehyde, 1R-α- pinene, 1S-α-pinene, octyl aldehyde, 6-butyl-1,4- cycloheptadiene, 6-hendecanone, 2-methoxylphenol, ( +)-aromadendrene, 1,4-cyclohexadiene-1,2-carboxylic acid, 3,4-dimethylepoxyphenylbutyrate) (↓) polysaccharide, ferulic acid, volatile constituents (ligustilide I, H, A, thujopsene, n-butylphthalide, α- and β- cedrene, n-butylidenephthalide, elemene, α-Chamigrene, α-bulnesol, β-chamigrene, ( +)-ledene, α-bisabolene, ( +)-cuparene, ( +)-α-longipinene, (-)-spainulenol, E- and Z- ligustilide)	(↑) hepatoprotective activity, anti-inflammation, antidiarrhea, spleen-tonifying (↓) intestinal irritation	(1) The decrease in sugar is attributed to the high temperature that causes it to carbonize; (2) Destruction of tissue structure and the loss of volatile oil; (3) Content of ferulic acid was decreased with the increase of temperature; (4) Increased solubility of chlorogenic acid and chlorogenic acid at high temperature; (5) High temperature could destroy enzyme activity and increase the content of pine ferulic acid ester. However, decompose could also be happen under too high temperature; (6) Senkyunolide I, senkyunolide H and n-butenylphthalide are unstable at high temperature	[75–77, 111, 112]
	Atractylodis Macrocephalae Rhizome	(↑) atractylenolide I,II, III, elemene, palmitic acid, linoleic acid, water soluble sugars and alcohol extract (↓) volatile oil (atractylone, geranione, etc.)	(↑) spleen-tonifying, antidiarrhea (↓) irritation	(1) Atractylone was oxidized to atractylodes by air contact oxidation; (2) Frying made the tissue loose and the volatile oil was lost; (3) Volatile oils were absorbed by soil	[78–80]
	Paeoniae Radix Alba	(↑) paeoniflorin, albiflorin, benzoic acid, gallic acid (↓) paeoniflorin	(↑) antioxidation, spleen-tonifying, antidiarrhea	(1) Paeoniflorin was decomposed into paeoniflorin under high temperature; (2) Heating changes content and structure of the water; (3) Trace elements in the soil played a synergistic role	[81, 82]
	Dioscoreae Rhizome	(↑) extract (water-soluble, alcohol-soluble) (↓) allantoin, polysaccharide, phospholipids	(↑) spleen-tonifying, antidiarrhea	(1) Loss of polysaccharide, phospholipid and allantoin by high temperature; (2) Synergetic effect of soil	[80, 85]
Soil	Coicis Semen	(↑) triglycerides	(↑) spleen-tonifying, antidiarrhea	(1) Frying made the tissue loose, while oil loss and effective components dissolved (2) Volatile components were absorbed by soil	[80]
	Atractylodis Rhizoma	(↑) trace elements (Fe, Cr, Ti, Al, Mn, Zn, B, Sn, P, Mo, Pt, Ni, Tl) (↓) trace elements (Cu, Na, Mg, Sr, Ba, Cd, Li)			[86]
	Citri Reticulatae Pericarpium	(↑) hesperidin	(↑) spleen-tonifying, dissipate phlegm, stop vomiting		[84]
	Lablab Semen Album	(↑) protein, total free amino acids, proline (↓) total phospholipids, total lectin	(↑) spleen-tonifying, antidiarrhea		[83]
	Myristicae Semen	(↑) volatile constituents (safrole, α-asarone, γ- and δ-terpinene, cis-p-menthane-2-ene-1-ol, α-phellandrene, β-caryophyllene, 4-carene) (↓) volatile constituents (myristol, trans-p-menthane-2- ene-1-ol, cis- and trans- β-terpinenol, β-pinene)			[46]
	Galli Gigerii Endothelium Corneum		(↑) digestion	(1) Stir frying with soil enhanced spleen-strengthening effect (2) Soil stir-fry correct heat, odor and flavor	[88]
	Eucommiae Cortex		(↑) spleen-tonifying, stop bleeding, liver-tonifying, kidney-tonifying	(1) Rubber filament is easy to break and absorption in salt water, which enhance the effect of tonifying liver and kidney (2) Soil could strengthen the spleen-strengthening and hemostasis effect	[87]
	Pinelliae Rhizoma	(↑) trace elements(Zinc, nickel) (↓) alkaloid (such as guanosine), lectin protein, calcium oxalate needle crystal	(↓) toxicity	Calcium oxalate and lectin protein were hydrolyzed and destroyed by lime water	[10, 91]
	Aristolochia Manshuriensis	(↓) aristolochic acid A	(↓) nephrotoxicity	Aristolochic acid A was neutralized and destroyed by alkaline substance	[60]
	Paridis Rhizoma		(↓) toxicity		[93]
Soda Lime	Alocasia Macrorrhiza		(↓) toxicity		[95]
	Strychni Semen	(↓) strychnine, brucine	(↓) toxicity		[95]
	Miao medicine Arisaema Rhizomatum	(↑) total flavonoids (processed with dry soda lime) (↓) total flavonoids (processed with soda lime water)			[92]
	Cordate Pinellia Tuber	(↓) alkaloid	(↓) toxicity	Acidic toxic component was hydrolyzed by alkaline soda lime water	[39]
	Indigo Naturalis	(↑) indigo blue, indirubin		Formation and enrichment of active components by the alkaline environment of lime water	[94]
Halloysitum Rubrum	Myristicae Semen	(↓) oil	(↑) antidiarrhea (↓) intestinal sliding	Antidiarrhea effect of absorbed oil by Red halloysite	[97]
	Atractylodis Macrocephalae Rhizome	(↑) polysaccharide, atractylenolide III	(↑) immunity, anti-oxidation, anti-cancer, anti-inflammatory		[98]
Cinnabaris	Ophiopogon Japonicus	(↓) total flavonoids	(↑) clearing the heart, calming the mind, aid-sleeping	Synergistic interaction	[103, 104]
	Polygalae Radix	(↑) cyclosenegenin	(↑) clearing the heart, calming the mind, aid-sleeping	Synergistic interaction	[102]

#### Arisaematis Rhizoma

Arisaematis Rhizoma, commonly known as ‘Tian-nan-xing’ in Chinese, is botanically from the dried tuber of *Arisaema erubescens* (Wall.) Schott, *Arisaema heterophyllum* Bl. or *Arisaema amurense* Maxim., which has the effect of dispersing knot and detumescence. While Tiannanxing is usually used to dry dampness and resolve phlegm, dispel wind and stop spasm as well as disperse knot and detumescence. However, its obvious toxicity was recorded in ancient books and reported by modern research. Dong et al. used the metabonomics method to reveal its nephrotoxicity mechanism. After intragastric administration of rat Tiannanxing (0, 0.5 and 1 g/kg body weight) for 30 days, the levels of serum urea nitrogen and creatinine were significantly increased. Significant changes of metabolites in blood and urine were also observed [108]. Similarly, another study revealed neurotoxic effects of Tiannanxing when given ICR mice for 3 days. However, it is worth noting that Tiannanxing that processed with alumen and ginger juice or bile not only reduced the neurotoxic effect but also enhanced the neuropharmacological effect [26]. After processing, the contents of amino acids and alkaloids are lower than that of raw products, which is similar to that of Banxia [27]. The content of inorganic elements zinc and calcium is lower than that of raw product, in return, the iron element obtained higher content, which indicates the introduce or loss of inorganic elements by processing [27]. At the same time, the agglutinin protein is degraded accompanied by the dissolution of toxic calcium oxalate needle crystal due to acid alumen water, and alumen is hydrolyzed into aluminum hydroxide to form gelatin, which reduce its toxicity by adsorption of toxic component owing to the electric charge [21–23, 28, 29].

#### Typhonii Rhizoma

Typhonii Rhizoma, commonly known as ‘Bai-fu-zi’ in Chinese, is botanically from the dried tuber of *Typhonium giganteum* Engl, which has been used to dispel wind and phlegm, calm convulsion, detoxification and relieve pain. However, its significant toxicity could not be ignored. Wang et al. treated RAW264.7 cells with different doses of Banxia lectin and found that inflammatory cytokines increased in a dose-dependent manner. Moreover, strong toxic effects related to inflammation were produced by long-term stimulation [30]. Compared with the raw materials, the contents of alkaloids, total saponins, nucleosides and other components of Baifuzi were changed after the process. Besides, new organic substances were generated, which promoted and strengthened the therapeutic effect of processed product. Significantly, the process of producing 5-hydroxymethylfurfural (5-HMF) by alumen water and heating could be attributed to the sugar thermal degradation and Maillard reaction of the sugars and amino acids of Baifuzi itself under the action of temperature and PH. Although 5-HMF has some toxic and side effects, it also possesses the effects of anti-oxidation and improving hemorheology, which increases the efficacy of Baifuzi in treating headache and vertigo of phlegm syncope. And 5-HMF was further transferred into bis (5-formylfuryl) ether by dehydration and condensation, which had antiviral, antioxidant and inhibitory effects on Mycobacterium tuberculosis. Meanwhile, the formation of above two organics has close association with the heating time and alumen consumption [31, 32].

The agglutinin protein contained in Baifuzi was degraded and denatured after being processed in alumen water [21, 22]. In addition, the loss of water-soluble free amino acids is relatively larger in the processing, the total amino acid content decreased by about 30% compared with the crude product, and the liposoluble components content increased, which was related to the alum fixation of protein and the promotion of lipid synthesis [32–35]. Meanwhile, the content of total alkaloids decreased greatly, which may be caused by the formation of soluble salt between acid alumen water and alkaloids [34, 36, 37]. Nucleosides, a class of biomolecules easily dissolved in water, their content was lower than that of raw product [34, 36]. Besides, the content of total flavonoids and saponins decreased slightly, while that of polysaccharides did not decrease significantly [36]. It is the same as Banxia processed with alumen, the aluminum content in Baifuzi processed with alumen is hundreds of times higher than that in raw one, which is due to the large amount of aluminum ions introduced from alumen [32, 34].

#### Others

Generally, alumen is mainly used for processing plants of the genus Araceae for reducing their toxicity. Apart from the three typical medicinal materials described above, the toxicity of Alocasia Macrorrhiza (Haiyu), the dried tuber of *Alocasia macrorrhiza* (L.) Schott, is also reduced due to the processing of alumen, while its antipyretic effect is weakened as well [38]. Cordate Pinellia Tuber (Dishuizhu), the dried tuber of araceae plant *Pinellia cordata* N. E. Br., another kind of Araceae plants, whose number of components with low polarity and chemical composition changed little after being processed by alumen. However, its high polar components decreased significantly due to promotion dissolution of small molecular compound with high polarity by the aluminium potassium sulphate in alumen [39].

Gardeniae Fructus (Zhizi), originate from the dry ripe fruit of *Gardenia jasminoides* Ellis, has also been reported to be processed with alumen. According to Tang’s study, the content of active ingredients such as total iridoid glycosides, geniposide, crocin I and crocin II extracted from Zhizi with six edges had been increased significantly after being boiled in alumen water [40]. Correspondingly, their curative effect was enhanced as well. However, there is some evidence that these ingredients will be reduced after being processed with alumen [41]. The divergence could be attributed to the different varieties or processing conditions. And all this has important significance to the improvement of the processing technology for the retention of active ingredients. In the literature and traditional processing methods of Zhizi, alumen water boilling has also been used to kill enzyme and reserve glycosides [41]. Furthermore, alumen could enhance the coloring of natural pigment, while the pigments of Zhizi could be widely used in food and textile industry [42]. Therefore, this method could reduce the environmental disruption to the quality of Zhizi due to the component loss caused by storage time.

### Talci pulvis

**Fig. 3:**
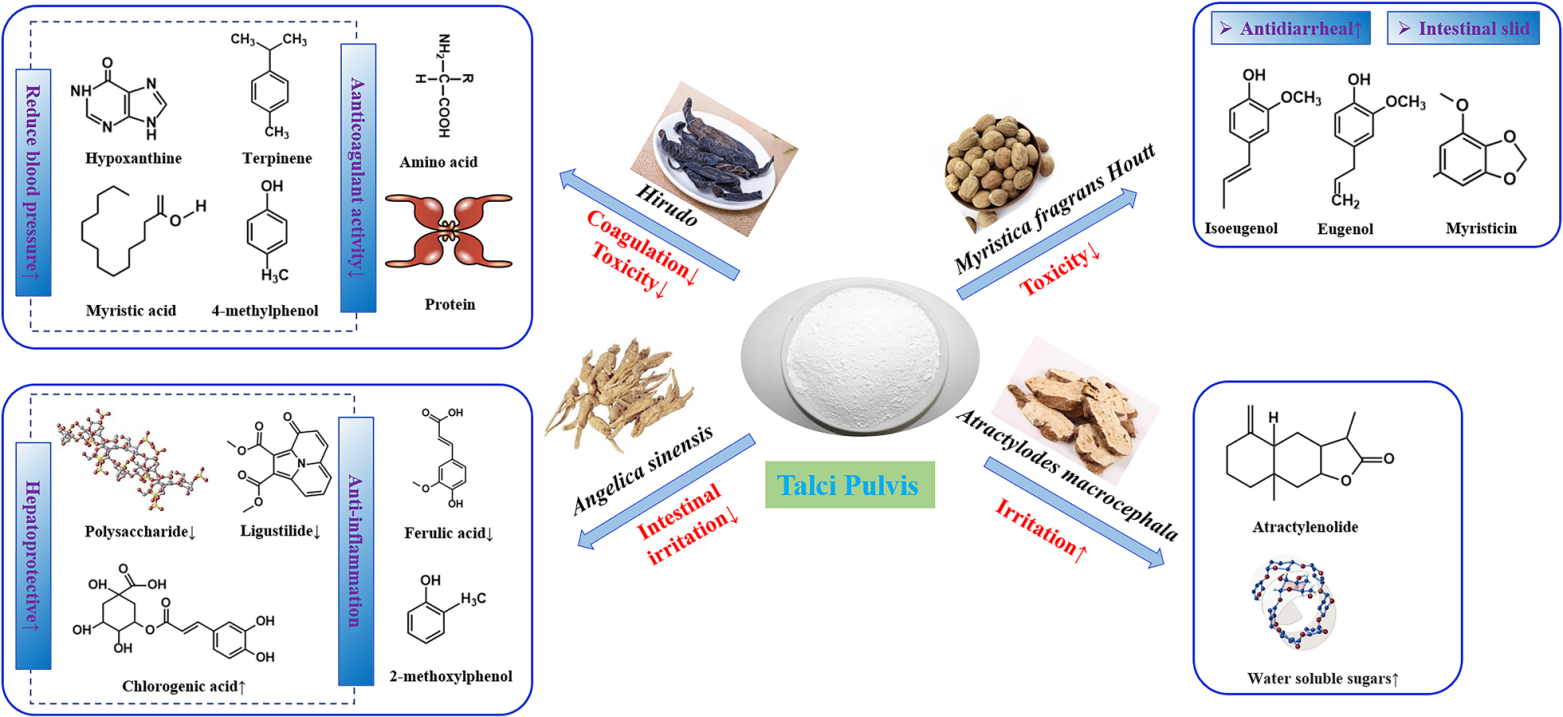
Effect of Talci Pulvis on the processing of traditional Chinese Medicine

Talci pulvis is widely used as a TCM, which possess the effect of diuresis, clearing away heat and detoxification, collecting dampness as well as astringent sores. Likewise, talci pulvis also plays an important role in the processing of TCM from three aspects. First, toxic and side effects of several toxic and irritating herbs including Roudoukou, Strychni Semen (Maqianzi), Aristolochia Manshuriensis (Guanmutong), Shuizhi and Bufonis Venenum (Chansu) could be reduced. Second, a synergistic action could be observed in some medicinal materials when processed with talci pulvis, such as Galli Gigerii Endothelium Corneum (Jineijin), Ejiao, Pheretima (Dilong). Third, some animal medicinal materials including swim bladder, corium elephatis and hedgehog skin were usually processed by talci pulvis in order to change the content of active ingredients and make the texture crisp and convenient for preparation (Fig. 3).

The application of talci pulvis in processing has been gradually developed according to the clinical requirement, although there were no relative records in ancient times. Most notably, the processing method of Hirudo with talci pulvis has been included in the ChP, National Standard for preparation of Chinese Materia Medica and the processing specifications of Guangxi, Henan, Heilongjiang, Jiangxi and other provinces. While that of Ejiao was recorded in the processing specifications of Hunan and Jiangxi Province [43, 44].

#### Myristicae Semen

Myristicae Semen, commonly known as ‘Rou-dou-kou’ in Chinese, is botanically from the seed kernel of *Myristica fragrans* Houtt. As a kind of major active component in Roudoukou, volatile oils bring us not only antibacterial, anti-inflammatory, anti-oxidation and other therapeutic effects but also intestinal sliding and toxic irritation. Although the raw material shows a variety of pharmacological activities, overdose can also lead to varying degrees of toxicity. Several literatures have reported that its toxic dose is 5 g, but some researchers have found that the 1–2 mg/kg of Roudoukou is enough to cause neurotoxicity. In addition, its hepatotoxicity, carcinogenicity and cytotoxicity that is closely related to its main active components (myristicin, safrole, engenol, elemicin, etc.) has also been reported [45]. Therefore, proper processing is beneficial for retaining its efficacy and reducing its stimulation. Among the constituents which contained in Roudoukou, monoterpenoids and aromatic compounds rank the first two place. Monoterpenoids were degraded or volatilized due to the high temperature with talci pulvis processing, while the content of aromatic compounds increased. In addition, destroyed hydroxyl structure and conversion into monoterpenes without oxygen atoms may lead to the decrease of efficacy [46]. As for the volatile oils, the content of myristicin, eugenol, isoeugenol, safrol and methyl eugenol was increased after being processed with talci pulvis [46, 47]. Among them, myristicin exhibited resistance against hepatic lipid peroxidation but hallucinogenic, eugenol and isoeugenol possessed antioxidant activity, safrol and methyl eugenol had carcinogenic and genotoxic effects. However, some studies showed that the content of myristicin decreased after processing with talci pulvis and that of safrol changed irregularly, which may be attribute to the variation in processing time and temperature [48, 49]. If the processing time was too short, the content of myristicin has instead increased [49]. The results indicated that the processing conditions showed great effect on its curative effect and toxicity.

Moreover, the property of talci pulvis is not consistent with the antidiarrheal effect of Roudoukou, so the efficacy, toxicity and other factors should be considered before we decide whether to use talci pulvis to process Roudoukou. It has been reported that the content of volatile oils in Roudoukou showed a trend of "first decreased and then rose" along with decreased particle size and increased dosage of talci pulvis [47]. These phenomena may be related to the heat transfer rate, particle size of powder, adsorption and other factors. While the stability of dehydroisoeugenol is less affected by these factors [47]. In addition to the effect on volatile oils, content of some inorganic elements such as Fe, Mn, Zn, Cu, Ca, Mg, Cr, Ni, Co and Po were also increased [50]. Briefly, appropriate processing conditions with talci pulvis could reduce the irritation and increase the content of effective components of Roudoukou, reasonable and unified processing technology that ensures the therapeutic effect need further study.

#### Toxic herbs

Some herbs containing toxic components were usually processed with talci pulvis for detoxification. Metal elements are widely regarded as toxic ingredients of Shuizhi, Pb, Cd and Hg contained therein were decreased after processing [51]. Hirudin, a major active ingredient of Shuizhi that affects its thrombolytic effect, its content was also reduced [51–53]. Instead, the content of amino acid in processed Shuizhi was obviously higher than that in raw products because proteins were more likely decomposed into small peptide and free amino acid at high temperature [51, 53, 54]. There are other studies showed that hypoxanthine increased significantly after processing [51, 53, 54]. Besides, some trace elements were also affected, the content of Ca and Mg increased, while that of Zn, Mn, Cu and Fe decreased [51]. Meanwhile, some volatile components were lost along with the produce of new ones [51, 55].

It is noteworthy that although the toxicity of Shuizhi is reduced due to the processing of talci pulvis, the efficacy of Shuizhi and some other toxic TCM were also affected accordingly. For example, bufogenin and resibufogenin in Chansu, strychnine and brucine in Maqianzi, which are all active but toxic, could be reduced after processed with talci pulvis [56–59]. Guanmutong, a controversial medicinal material with severe nephrotoxicity, had been removed from the ChP, because of its toxic component aristolochic acid A, which can be destroyed about 40% by the high temperature processing of talci pulvis [60]. Therefore, the appropriate technology for reducing the toxicity and retaining the efficacy when processing toxic herbs with talci pulvis should be given in the future research.

#### Others

In addition to reducing toxicity, enhancing the efficacy is also a mechanism of talci pulvis application. For example, a synergistic role could be observed when Jineijin and Pheretima was used for stranguria, heat-clearing and diuretic, respectively [61, 62]. The reason why Jineijin is used for digestion after processed with talci pulvis is probably related to the increase of protease activity, but the amylase activity is lower than that of raw product [62]. It can be inferred that processed Jineijin is more suitable for the indigestion that caused by eating too much food with high protein content. Besides, the content of total and essential amino acids in Ejiao increased apparently after processing with talci pulvis, along with its enhanced hemostatic and blood-enriching effect [63]. The mechanism may be associated with the magnesium silicate contained in talci pulvis, which not only promote the produce of a large amount of colloidal protein and calcium salt but also enhance the present convergent, absorption, promoting blood coagulation and reducing bleeding effect [64].

When talci pulvis is used as a processing auxiliary material, it acts as an intermediate heat transfer body with strong fluidity and fine powder, which is suitable for processing animal medicinal materials with strong toughness. The smashing rate and protein content of the swim bladder are closely related to the dosage of talci pulvis. Generally, more talci pulvis is associated with fuller swim bladder glue swell, higher grinding efficiency and more preserved protein [65]. After processing, some new volatile components such as 3,4,5-trimethoxytoluene, α-gurulene, 3-methyl biphenyl and caryophyllene oxide were produced by the swim bladder and hawksbill shell along with the loss of volatile components 2-decennal, myristic aldehyde, cedarene and valenciennes [55]. Moreover, the content of amino acids in corium elephatis increased after processing, while that in dog testes and penis and hedgehog skin decreased, which was related to heating time and temperature [66–68]. It is also noteworthy that these animal medicinal materials are not commonly used in clinical in view of wildlife conservation, although included in some local standards. In general, we used this method to process animal medicinal materials mainly because we can mask the disagreeable odors and make them crisp for decocting the active ingredients. There are also other examples that processed with talci pulvis. For example, the effect on invigorating spleen and relieving diarrhea of Puerariae Lobatae Radix (Gegen) is strengthened after processing with talci pulvis. The phenomenon is mainly resulted from the increased content of total flavonoids [69]. Moreover, processing these herbs with talci pulvis remain one of the most popular approaches recorded in some provincial processing standards, although have not yet been included in ChP.

### Soil

**Fig. 4:**
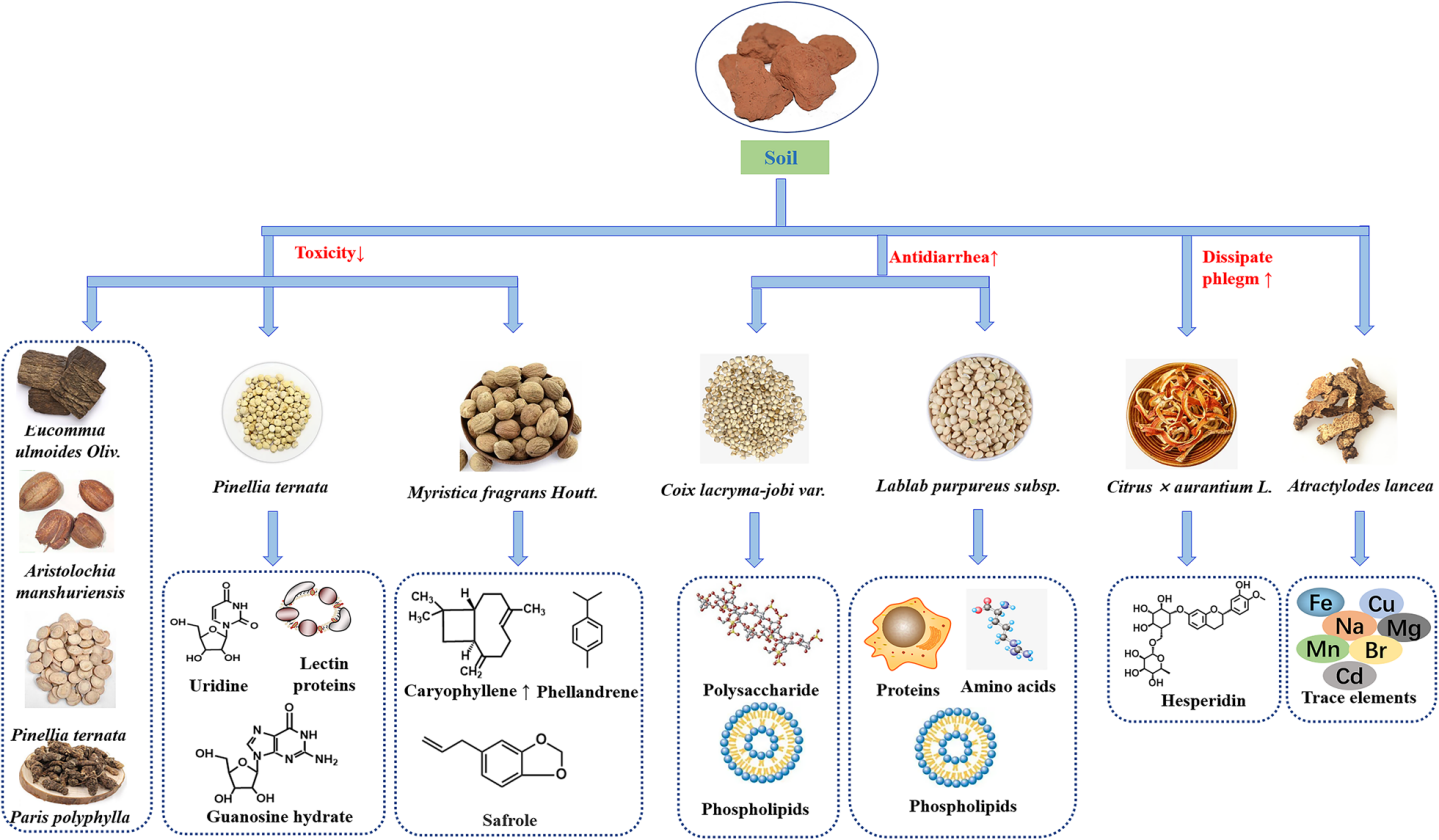
Effect of Soil on the processing of traditional Chinese Medicine

In traditional Chinese medicine processing, the soil could be used as medicine to stop bleeding, nausea and diarrhea. Normally, the soil we use is the burnt loess in the stove where farmers burn firewood. We call it “Zao-xin-tu”, also known as “Fu-long-gan”. Many alternatives are virtually used due to its rareness. After being roasted in the soil, many medicinal materials possessed the functions of soothing the stomach, stopping vomiting and diarrhea as well as reducing gastrointestinal irritation. The main reason is that the mineral and inorganic salts of the soil could be decomposed into a variety of basic oxides after being heated and refined at high temperature, which neutralized gastric acid and play a role of heat conduction as well [70]. In general, drugs with spleen-tonifying and antidiarrheal effects need to be stir-fried with soil, such as Danggui, Baizhu, Baishao, Yiyiren, Dioscoreae Rhizoma (Shanyao) and so on (**Fig. ****4**).

Products of Danggui, Baizhu, Baishao, and Yiyiren that stir-fried with soil were recorded in historical document *Bencaohaili(《本草害利》), Depeibencao(《得配本草》)* [71], *Qianjinyifang (《千金翼方》)* [72], *Bencaomengquan(《本草蒙筌》)* [73] and *Bencaocongxin(《本草从新》)* [74], respectively. And they were also included in the ChP and some local regulations as shown in Table 1.

#### Angelicae Sinensis Radix

Angelicae Sinensis Radix, commonly known as ‘Dang-gui’ in Chinese, is botanically from the dried root of umbelliferae plant *Angelica sinensis* (Oliv.) Diels. It could be used to nourish blood, promote blood circulation, regulate menstruation, relieve pain, moisten intestines and defecation. Volatile oils served as a kind of its major active component. After stir frying, the volatile components of Danggui, such as ligustilide, senkyunolide I, senkyunolide H and senkyunolide A, were destroyed and lost due to high temperature [75]. Yet it's worth noting that part of the efficacy was weakened by the reduction of volatile oils when compared with crude herbs, irritation of medicinal materials was also alleviated. Besides, the content of chlorogenic acid increased due to its improved solubility in organic solvent at high temperature, while that of ferulic acid decreased on account of the thermal instability [75]. As for coniferyl ferulate, whose content was increased because the activity of its degrading enzyme was destroyed with the increase of temperature [75].

Angelica sinensis polysaccharide, a kind of water-soluble macromolecular component, has the effects of improving blood system, promoting immunity, anti-tumor and anti-radiation. After processing, the content of water-soluble sugar, reducing sugar and polysaccharide in Danggui was lower than that in raw product because of carbonization by high temperature, but the decrease was not significant [76]. In addition, formic acid, acetic acid and 1-ethoxypropane showed a relative upward tendency, while N-butylphthalide and N-butenylphthalide decreased significantly [77]. Some studies showed that the content of volatile oil in Danggui decreased significantly after being processed with soil, which could alleviate the irritant intestines, reduce the toxic and side effects, and improve its spleen-tonifying, antidiarrhea, anti-inflammation and hepatoprotective activity [78, 79].

#### Atractylodis Macrocephalae Rhizoma

Atractylodis Macrocephalae Rhizoma, commonly known as ‘Bai-zhu’ in Chinese, is botanically from the dried rhizome of compositae plant *Atractylodes macrocephala* Koidz., which could strengthen spleen, replenish Qi, dry dampness and promote diuresis. Atractylenolides, atractylone, volatile oils and polysaccharides are the major active components of it. After processing with soil, the content of atractylenolide I, II and III increased, while that of atractylone decreased [78]. The main reason is that Baizhu became loose after crushing, atractylone was oxidized into atractylenolide I, III and biatractylolide by contacting with oxygen, and atractylenolide III was further transformed into atractylenolide II during the heating dehydration process [79, 80]. Meanwhile, the decrease of atractylone contributed to reduced stimulation [79]. The content of polysaccharide, the main component of invigorating the spleen and stopping diarrhea, had no significant change, while the content of water-soluble sugar increased significantly. Similarly, other components such as elemene, palmitic acid and linoleic acid increased significantly [78].

#### Paeoniae Radix Alba

Paeoniae Radix Alba, commonly known as ‘Bai-shao’ in Chinese, is botanically from the dried root of ranunculaceae plant *Paeonia lactiflora* Pall..It could be used to nourish blood and regulate menstruation, astringe Yin and stops weating, soften liver and relieve pain as well as stabilize liver yang. Paeoniflorin and albiflorin, two major ingredients of Baishao recorded in ChP, were slightly increased after being fried in soil [80]. There were other studies, though, which reported the content of paeoniflorin was lower than that of raw products [81, 82]. The phenomenon was caused partly by the decomposition of paeoniflorin into albiflorin under the catalysis of high temperature conditions and auxiliary ingredients [81]. Therefore, different processing conditions lead to the different contents of active ingredients. Recently, some scholars compared the antioxidant capacity of raw and processed products of Baishao. The antioxidant capacity of processed one is stronger in view of the increased gallic acid and benzoic acid after soil processing [82]. Likewise, spleen-strengthening and anti-diarrhea effect of Baishao was also enhanced. This is a result for the one side from the assist of the effective components by the trace elements in soil, and for the other side from the its own hemostasis and anti-vomiting characteristics of soil during cooling and anti-diarrhea process [80].

#### Others

Process with soil usually changes the active ingredients and enhance the curative effect of some TCM with spleen-strengthening activity. Take Yiyiren as an example, its dissolution of oil components and the content of triglyceride was increased due to the looser texture after the process with soil [80]. Moreover, the relative increased lactones and changed the oil composition was also observed owing to the absorption effect of soil. As for Lablab Semen Album (Baibiandou), the content of protein and free amino acid were increased accompanied by the decrease of total phospholipid and lectin after process [83]. Accordingly, its effect on regulating gastrointestinal hormone level, strengthening spleen and stopping diarrhea was enhanced. There are also some reports of Chenpi that have been processed with soil, whose content of hesperidin was increased [84]. However, content of polysaccharide, allantoin and phospholipid of Shanyao decreased because of the high temperature [80, 85]. Apart from the above cases, trace elements in Atractylodis Rhizoma (Cangzhu) were also changed. The levels of Fe, Cr, Ti, Al and TL increased by 2 ~ 4 times, while that of Cu, Na, Mg, Sr, Ba, Cd and Li decreased by 2–3 times [86]. Besides, the content of aromatic compounds in Roudoukou changed greatly, except for the decreased myristate and increased safrole, a new component α-asarone was produced accordingly [46]. Besides, soil process could make the rubber filament of Eucommiae Cortex (Duzhong) easier to break and improve the peculiar smell of GGEC [87, 88].

### Soda lime

**Fig. 5:**
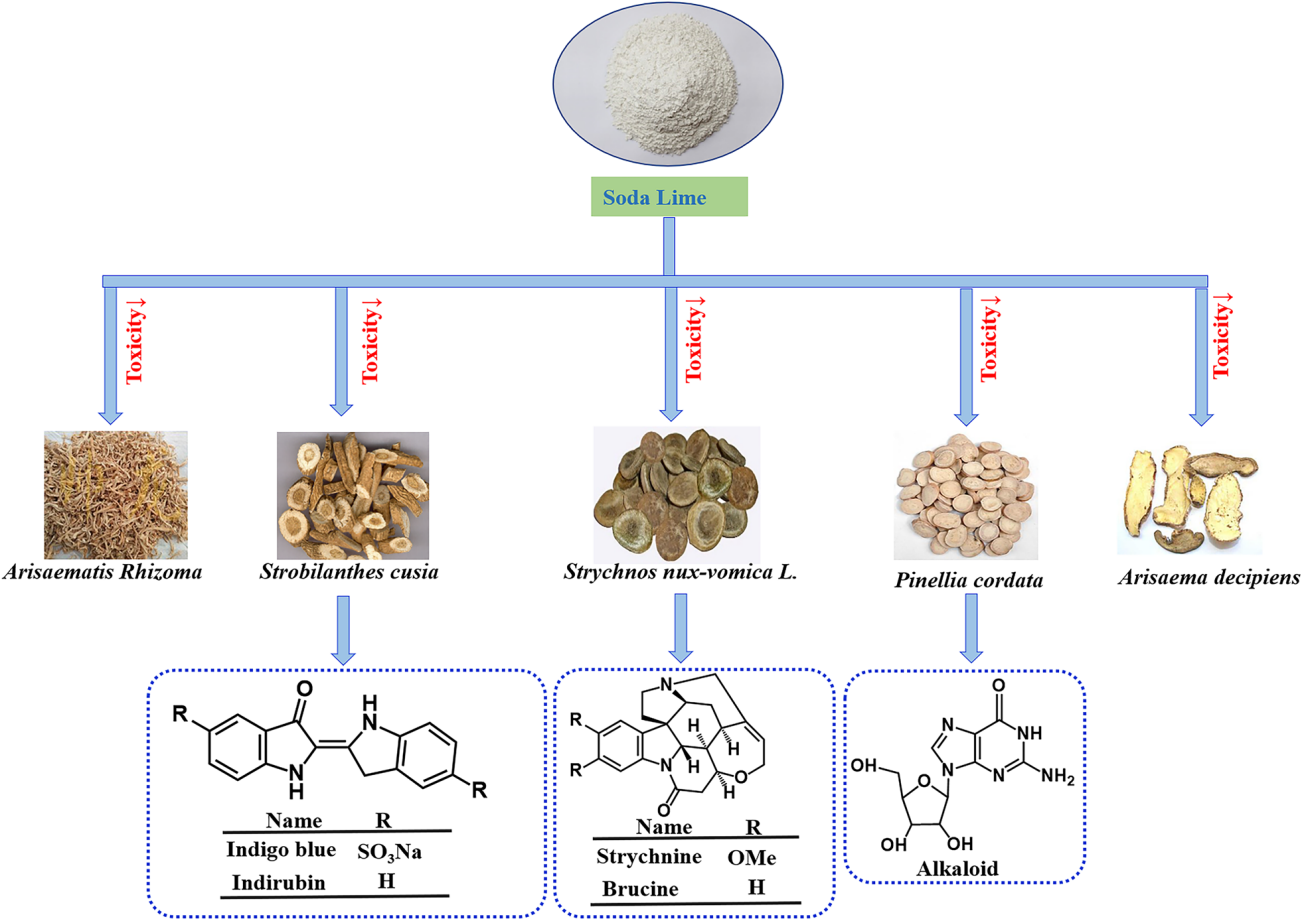
Effect of Soda Lime on the processing of traditional Chinese Medicine

Soda lime is a mixture of calcium hydroxide, sodium hydroxide or potassium hydroxide, which could also be used as pharmaceutical excipients that reduces toxic components in the processing of TCM. The medicinal materials processed with soda lime are Guanmutong, Banxia and other araceae plants (Fig. 5). The processing of Banxia with soda lime water can be traced back to the Ming Dynasty, which is recorded for the first time in the *Yaoxingcuping(《药性粗评》)*. In addition, they were also mentioned *in Yaoxinghuiyuan(《药性会元》)* and *Bencaogangmushiyi(《本草纲目拾遗》)* [5]. At present, the pharmaceutical standard of soda lime water-processing Banxia has been included in the ChP. The processing method of Tiannanxing with soda lime water can be found in the work *Xianshoulishangxuduanmifang(《仙授理伤续断秘方》)* of the Tang Dynasty[89]. SS processed with soda lime water is a folk method. When used in Yangjiang area, the toxicity of it can be greatly reduced [90].

The detoxification mechanism of processing Banxia by soda lime water was similar to that of processing it with alumen. Moreover, the alkaloids and proteins, two kinds of effective and toxic components in Banxia, were also decreased [10, 91]. The change of trace elements is illustrated by the increase of Zn content and production of new element Ni [10]. Miao medicine Arisaema Rhizomatum (Banjielian), which is the dried tuber of araceae plant *Arisaema rhizomatum* C.E.C. Fischer. When processed by soaking or steaming in soda lime water, the content of total flavonoids in Banjielian decreased significantly, while that was increased when dry soda lime was used [92].

Dishuizhu has strong irritation to the mucous membrane of eyes, throat, intestines and stomach, so its processed products are used in clinic for its lower toxicity and resistance. After processed with soda lime water, the number and content of the chemical components in Dishuizhu were both affected, and the decreased content of total alkaloids, uridine and adenosine in which is most obviously [39]. The reason for the results may be that toxic components were mainly small molecules of acidic components and could be removed by the neutralization with soda lime water. In alkaline solution, meanwhile, the loss of uridine and adenosine is large [39].

As we mentioned above, aristolochic acid A is the toxic component of Guanmutong, which was usually processed with talci pulvis. Significantly, its toxicity could also be reduced by boiling or steaming with soda lime water in view of the reaction with strong alkali supported by soda lime water [60]. Soda lime water can also reduce the toxicity of other herbs, such as Paridis Rhizoma (Chonglou), Haiyu, and Maqianzi. However, their detoxification mechanism is still unclear, which needs further study [93, 94]. Promoting the formation of active ingredients is another processing mechanism of soda lime water. Indigotin, an active component of Indigo Naturalis (Qingdai) was formed and enriched by the promotion of soda lime water through providing alkaline environment, CO_2_ and carrier [94].

Some literatures also mentioned the enhanced antidiarrheal and anti-inflammatory effect of guava leaf and holly leaf and reduced toxicity of Huechys (Hongniangzi) by processed with soda lime. Besides, soda lime could also prolong the storage life of Dilong by providing strong alkaline and hygroscopic environment [95].

### Halloysitum Rubrum

Halloysitum rubrum is a silicate mineral, which has the effect of astringent intestines, hemostasis, myogenesis and sores, and is often used as a processing excipient for soil frying in the process of TCM processing [96].

Roudoukou could be processed by halloysitum rubrum for both reducing the side effects of diarrhea caused by absorption of oil and increasing the antidiarrheal effect [97]. Likewise, immunity, anti-oxidation, anti-inflammatory and anti-cancer effects of Baizhu were also enhanced by halloysitum rubrum for the increased effective components atractylenolide III and polysaccharide, especially the atractylenolide III, whose content was about 3–4 times higher than the raw product [98].

### Cinnabaris

Cinnabaris is a kind of sulfide mineral medicine, which has the effects of clearing the heart, calming the mind, brightening the eyes and deintoxication, but is toxic. Cinnabaris is usually prepared by elutriation to reduce the content of sulfur and calcium, so as to reduce toxicity and improve cleanliness. Although the toxicity of cinnabaris is reduced after processing, it is rarely used as an excipient when processing TCM due to the dosage that need to be controlled. The method of processing Maidong with cinnabaris was recorded in the reading of *Bencaobiandu(《本草便读》)* in the Qing Dynasty, and the use of cinnabaris mixed Maidong appeared in *Wujutongyian(《吴鞠通医案》)* and *Bencaohaili(《本草害利》)* for the first time[99]. There is little discussion on the tracing of ancient books on processing Yuanzhi with cinnabaris, but some documents mention that this processing method has been used since ancient times and is still in use at present [100, 101].

Generally, it could be used to process some TCM with anti-anxiety and psychotherapeutic activity on the premise of paying attention to the usage and dosage. Studies had shown that, after processing, the content of cyclosenegenin in Yuanzhi was 6 times higher than that of the raw product, while that of senegenic acid and senegenin was basically unchanged [102]. Some studies have questioned the method of processing Maidong with cinnabaris [103], mainly because of its toxicity and decreased total flavonoids after process [104]. Overall, the processing technology of cinnabaris used for preparing TCM should be further standardized in order to ensure the efficacy and reduce the side effects, which realize its full synergy potential.

### Future perspective

To sum up, mineral excipients play an irreplaceable role in the processing of TCM. Among them, alumen and soda lime mainly work at reducing the toxicity of the main drug, but the effective components will also be affected, and thus bring weaker curative effect than that of raw products. Talci pulvis are usually used for processing some medicinal materials containing volatile oils, which relieve gastrointestinal irritation under the premise of ensuring the efficacy and degrade the toxic components of some toxic medicinal materials under high temperature. In addition, some animal medicinal materials could also be deodorized and flavored after processing with talci pulvis, which make their texture crispy and convenient for dispensing. Soil possessed anti-emetic, astringent and hemostatic effects for gastric. It is generally used as auxiliary materials to process herbs with spleen-strengthening and antidiarrhoeal effects and the mechanism was related to enhancing the efficacy and alleviating the irritation through changing the content and composition of volatile oils in herbs. Halloysitum rubrum, as an alternative to the soil, exhibited astringent intestines and antidiarrheal activity by reducing the oil content of medicinal materials. Cinnabaris is an auxiliary material that enhances the heart-clearing and sedative effect of herbs and should be used cautiously due to its toxicity. However, due to their own particularity and affect by various factors, investigated in many aspects including processing technologies, quality standards of mineral excipients and processing mechanism need further study and discuss.

- Standardization of processing methods
  The processing methods including processing temperature, time, acid–base environment and dosage of excipients need to be standardized, so that the effective components of medicinal materials could be fully retained without increase or decrease inconsistently, which greatly ensures the effectiveness of medicinal materials. Besides, the toxic components of many drugs are also active ingredients. How to retain the maximum effectiveness and reduce the harmfulness as much as possible needs to start from the processing technology for unified specification. Standardization of processing technology can better control the quality standard of processed medicinal materials.
- Quality control of mineral excipients
  Some mineral excipients such as cinnabaris are toxic or harmful to the human body. Hg^2+^, HgCl, Hg (CH_2_COOH)_2_ and other trace elements such as Ba, Sb are the main toxic components of cinnabaris [105]. Their metabolism is slow and easy to cause heavy metal accumulation, resulting in liver and kidney injury. Moreover, aluminum accumulation of alumen will affect the development of bone cells [106] and could even lead to visual, memory and other neurological disorders like Alzheimer's disease [107]. Thus, their content, purity and dosage are necessary to control when used as excipients, which help to reduce the loss of effective ingredients and residues of harmful substances.
- In-depth study on processing mechanism
  For now, the variety in the content and composition of active ingredients from many herbs before and after excipients processing is still unclear with inferred results. Significantly, the mechanism research should be combined with pharmacological research in order to explore which are the active ingredients, how to reduce component loss and toxicity as well as better processing technologies.
- Study on the processing of medicine food homology plants
  Some medicine food homology plants including Shanyao, Yiyiren and Baizhu were usually fried with soil. After processing, their spleen-strengthening and antidiarrhoeal effects on stomach had been increased. When used as food, however, whether their soil fried products with increased efficacy could be used and how to define the quality standards need in-depth discussion.

## Conclusion

The processing of TCM has received more and more attention with the development of analytical technology and toxicological research. As an important part of the processing field, the mineral excipients have played an essential and indispensable role in TCMs application. In this review, the significance of mineral excipients including alumen, talci pulvis, soil, soda lime, halloysitum rubrum and cinnabaris on TCMs has been summarized from the insight into the components, pharmacodynamics and mechanism, which not only supply comprehensive information on how to balance the necessity and relative merits and disadvantages of mineral excipients in clinical application, but also provide a reference for exploring better processing excipients and technology so as to reserve the efficacy as well as reduce toxic and side substances to the human body.

## Data Availability

Not applicable.
