# Correction to: Effect of mineral excipients on processing traditional Chinese medicines: an insight into the components, pharmacodynamics and mechanism

**DOI:** 10.1186/s13020-021-00566-4

**Published:** 2022-01-06

**Authors:** Yan Liu, Xiaojie Li, Cai Chen, Aijing Leng, Jialin Qu

**Affiliations:** 1 Department of Traditional Chinese Medicine, The First Affliated Hospital of Dalian Medical University, No. 222, Zhongshan Road, Dalian, 116011 China; 2 Clinical Laboratory of Integrative Medicine, The First Affliated Hospital of Dalian Medical University, No. 222, Zhongshan Road, Dalian, 116011 China; 3 Institute (College) of Pharmacy, Dalian Medical University, No. 9, South Road of Lvshun, Dalian, 116044 China; 4 Institute (College) of Integrative Medicine, Dalian Medical University, No. 9, South Road of Lvshun, Dalian, 116044 China

## Correction to: Chinese Medicine (2021) 16:143 https://doi.org/10.1186/s13020-021-00554-8

Following publication of this article [1], the authors would like to change the order of the affiliations 2 and 3. The correct order is given in this article.

^1^Department of Traditional Chinese Medicine, The First Affliated Hospital of Dalian Medical University, No. 222, Zhongshan Road, Dalian 116011, China.

^2^Clinical Laboratory of Integrative Medicine, The First Affliated Hospital of Dalian Medical University, No. 222, Zhongshan Road, Dalian 116011, China.

^3^Institute (College) of Pharmacy, Dalian Medical University, No. 9, South Road of Lvshun, Dalian 116044, China.

^4^Institute (College) of Integrative Medicine, Dalian Medical University, No. 9, South Road of Lvshun, Dalian 116044, China.

The original article [1] has been corrected.

## References

[1] Liu Y, Li X, Chen C, Leng A, Qu J (2021). Effect of mineral excipients on processing traditional Chinese medicines: an insight into the components, pharmacodynamics and mechanism. Chin Med.

